# The Smallest Form Factor UWB Antenna with Quintuple Rejection Bands for IoT Applications Utilizing RSRR and RCSRR

**DOI:** 10.3390/s18030911

**Published:** 2018-03-19

**Authors:** MuhibUr Rahman, Jung-Dong Park

**Affiliations:** Division of Electronics and Electrical Engineering, Dongguk University, Seoul 04620, Korea; muhib@dongguk.edu

**Keywords:** UWB antenna, rejection bands, rectangular complementary split ring resonator (R-CSRR), rectangular split-ring resonator (RSRR), Internet of things (IoT)

## Abstract

In this paper, we present the smallest form factor microstrip-fed ultra-wideband antenna with quintuple rejection bands for use in wireless sensor networks, mobile handsets, and Internet of things (IoT). Five rejection bands have been achieved at the frequencies of 3.5, 4.5, 5.25, 5.7, and 8.2 GHz, inseminating four rectangular complementary split ring resonators (RCSRRs) on the radiating patch and placing two rectangular split-ring resonators (RSRR) near the feedline-patch junction of the conventional ultra-wideband (UWB) antenna. The design guidelines of the implemented notched bands are provided at the desired frequency bands and analyzed. The measured results demonstrate that the proposed antenna delivers a wide impedance bandwidth from 3 to 11 GHz with a nearly omnidirectional radiation pattern, high rejection in the multiple notched-bands, and good radiation efficiency over the entire frequency band except at the notched frequencies. Simulated and measured response match well specifically at the stop-bands.

## 1. Introduction

Since 2002, the Federal Communication Commission (FCC) has permitted its commercial ultra-wideband (UWB) systems to operate within the 3.1 GHz to 10.6 GHz frequency range [1,2]. UWB technology has attracted much attention from researchers because of wide impedance bandwidth, high-speed data rate, and good time domain resolution [3,4]. In sensing applications, UWB antennas are used in short-range transmission [5], medical applications [6,7], surveillance systems [8], Internet of things (IoT) [9], and wireless body area networks (WBANs) [10]. In medical sensing applications and microwave imaging sensing, UWB radar has been in the spotlight owing to its high accuracy and robustness in a multipath environment. The authors of [6] implemented UWB sensors for detecting microscopic malignant breast tumors and checked their feasibility using multilayered breast phantoms. The authors of [8] designed two different UWB antennas for the solution of pulsed asset tag devices. For portable IoT sensors and WBANs, the compact-sized antenna has received much interest, and can be easily embedded in IoT devices [11,12,13]. However, owing to the extremely broadband of operation for UWB systems, there exists an inevitable overlap between UWB communication systems and many narrowband wireless communication systems such as Worldwide-Interoperability for Microwave-Access (WiMAX) operating at 3.30–3.60 GHz, the Indian National Satellite (INSAT) operating at 4.50–4.80 GHz, the wireless local area network (WLAN) operating at 5.15–5.35 GHz and 5.725–5.825 GHz, X-band satellite communication operating at 7.2–7.65 GHz, and the International Telecommunication Union (ITU 8-GHz) frequency band operating at 7.95–8.55 [14]. Hence, we aim to design a compact-sized antenna for IoT applications which can achieve multiple-notched bands to avoid the potential interferences caused by these narrow-band communication systems within the UWB frequency band [15]. Also, the antenna structure should be simple enough to be fabricated at low cost.

The concept of introducing frequency notched bands within UWB antennas was initially proposed in [16,17]. After these early works, different methods and techniques were developed for designing band-notched UWB antennas, including tuning stubs, the addition of parasitic resonators [18,19,20,21], and etching slots [22,23,24,25,26,27,28,29]. In other works [22,23,24], single band-notched UWB antennas were reported for suppressing interference from the WLAN frequency band. Dual band-notched UWB antennas were reported in [25,26,27,28,29] to avoid the interference from both the WiMAX and WLAN frequency bands. However, all of these antennas have only single and dual notched bands. Moreover, the frequency selectivity of the embedded notch is not good enough that it rejects the entire WLAN (5–6 GHz) frequency band, though the desired rejection bands are from 5.15 to 5.35 GHz for lower WLAN band and the upper WLAN frequency band operating at 5.725–5.825 GHz. Thus, any useful data within the range of 5.35–5.725 GHz will also be lost, resulting in the severe degradation of the quality of the received UWB signal.

To reject the lower and upper WLAN frequency bands along with the WiMAX frequency band, researchers had worked on triple band-notched UWB antennas. However, designing triple band-notched UWB antenna is a difficult task since controlling the notched frequency bandwidth in a limited space creates an undesired and complicated coupling between the resonators. In [29], the authors achieved a sharp rejection of the upper and lower WLAN frequency bands by using independently controllable strips. In [30], a combination of complementary split ring resonators (CSRRs) and DGS resonators was used to achieve the tri-band notching behavior with rejection frequency bands at 3.3–4.0, 5.15–5.40, and 5.80–6.15 GHz. In [31], the authors achieve tri-band notching at 3.40–3.65, 4.90–5.20, and 5.30–5.85 GHz by introducing two C-shaped slot resonators on the radiating patch and symmetric C-shaped slot resonator pair on the modified ground plane. By implementing the hook-shaped Defected Ground Structure (DGS) with the combination of slot resonators on the patch as well as ground plane, a triple band-notched band antenna is also reported in [32]. Similarly, a pair of CSRRs was used in [33] to achieve the triple band rejection in the frequency bands of 3.3–3.6, 5.1–5.4, and 5.7–5.9 GHz. In [34], they achieved the triple band notching in the frequency bands centered at 3.50, 5.50, and 8.10 GHz by introducing two rectangular complementary split ring resonators (RCSRRs) at the junction of the feed line, a meander-shaped stub, and an inverted U-shaped slot on the center of the radiating patch. Despite the recognition of different triple band-notched UWB antennas proposed in the literature, these designs have drawbacks in the sense of large dimensions, incomplete rejection of the interfering bands, and irregular and complicated resonator structures such as a combination of stubs and slot resonators. Also, triple band-notching is not enough to reject all the interfering bands as UWB have a broad spectrum, and other frequency bands such as INSAT (4.5–4.8 GHz), X-band satellite communication (7.2–7.65 GHz) and ITU 8-GHz (7.95–8.55 GHz) also fall.

In this context, quadruple band-notched UWB antennas have been proposed to mitigate the potential interferences at four different bands. A quadruple band-notched UWB antenna has been proposed in [35] that has the capability to reject 2.3–2.9, 3.2–3.7, 5.2–5.89, and 8.06–8.8 GHz frequency bands. In [36] the authors designed a quad-notched UWB antenna on multilayer surfaces to reject the WiMAX, INSAT, lower and upper WLAN frequency bands. Similarly, in [37] the authors designed a quadruple band-notched antenna by the combination of C-shaped and Nested C-shaped slot resonators in the patch with the addition of U-shaped slot resonators in the ground plane. The antenna has the capability of rejecting 2.4–2.7 GHz, 3.42–3.97 GHz, 5.4–6.0 and 8–8.68 GHz, for notching WiMAX, WLAN, and ITU 8 GHz frequency bands. In [38], they designed a quad-notched UWB antenna for rejecting the WiMAX, INSAT, lower and upper WLAN bands and analyzed CSRR to CSRR coupling among them. After this work, researchers investigated the quintuple band-notching technique within the UWB frequency bands in order to further minimize the jamming effect within the UWB frequency band. In this regard, the first attempt to design quintuple band-notched antenna was carried out in [39]. Although the antenna successfully rejects the five narrow frequency bands, the reported antenna size is quite bulky (80 × 80 mm^2^) for use in portable IoT applications. Similarly, the authors of [40,41] proposed a quintuple band-notched UWB antenna which has a capability to reject 3.6, 5.2, 5.8, 7.5, and 8.3 GHz frequency bands. However, they have placed the five slot resonators in a very irregular pattern which makes it very tough to judge the exact position of the notches in their design. Also, the frequency response of the notched bands is not steep enough (with a Voltage Standing Wave Ratio (VSWR) between 2.2 and 3.8) to achieve desirable rejection performance.

In this paper, we present a microstrip-line fed planar quintuple ultra-wideband (UWB) antenna using a combination of the rectangular complementary split ring resonator (RCSRR) and the rectangular split-ring resonator (RSRR) for rejecting the WiMAX, INSAT, lower WLAN, upper WLAN, and ITU 8 GHz frequency bands. At the beginning, a conventional stair cased structured reference UWB antenna is designed and fabricated to operate within the desired UWB frequency band. Then, the reference UWB antenna is modified for the proposed quintuple band-notched UWB antenna to reject the 3.5, 4.5, 5.25, 5.7, and 8.2 GHz bands for WiMAX, INSAT, lower WLAN, upper WLAN, and ITU 8 GHz frequency bands, respectively. To optimize the dimensions of the RSRR and RCSRR, a commercially available 3D-EM software, Ansoft High Frequency Structure Simulator (HFSS) is used. The proposed antenna with quintuple rejection bands is fabricated, and the measurements are presented for the verification. Finally, the design process in relation to the reflection coefficient, VSWR, antenna gain, radiation efficiency, and radiation pattern are described in detail. The proposed antenna performance has been checked for tumor detection, and the simulated results are presented.

The geometrical analysis and simulation results of the antenna are discussed in detail in Section 2 with corresponding results. The measurement setup and experimental results of the proposed and the reference UWB antenna are explained in Section 3. Section 4 deals with the comparison of the proposed antenna with another recent state-of-the-art design reported in the literature. Section 5 provides the application of the proposed antenna in microwave sensing, and the conclusion then follows.

## 2. Design and Simulation Results

**Figure 1:**
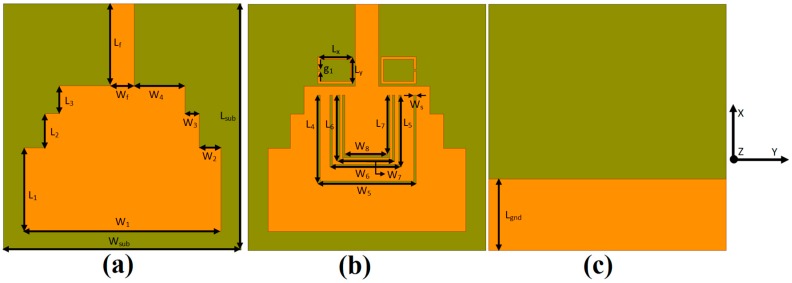
The geometry of (**a**) the reference antenna (front view); (**b**) the proposed antenna (front view); and (**c**) the reference and proposed antenna (rear view).

**Figure 2:**
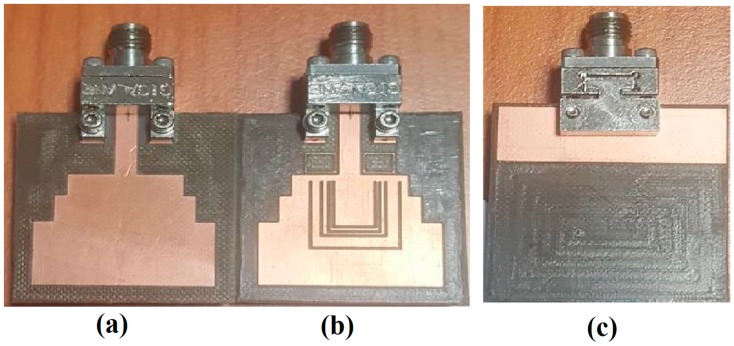
Prototype of the reference and proposed antenna. (**a**) The reference antenna (front view); (**b**) the proposed antenna (front view); and (**c**) the reference and proposed antenna (rear view).

Figure 1a,c shows the front and rear-view geometry and dimensions of the reference UWB antenna while Figure 2a,c shows the front and rear view prototype of the conventional UWB antenna designed [35] and termed as a reference UWB antenna. This antenna was designed on RogersRO5880 substrate with a thickness of 31 mils, a relative dielectric constant of 2.2, and a loss tangent of 0.0009.

A modification was carried out for the reference UWB antenna in order to efficiently reject the interfering frequency bands that fall within the UWB frequency band. Figure 1b,c show the front and rear-view geometry and dimensions of the proposed quintuple band-notched UWB antenna. The prototype of the proposed antenna is also fabricated as shown in Figure 2b,c. The dimensions of the reference and proposed antenna is listed in Table 1.

### 2.1. Design Equations for the RCSRR and RSRR

For the initial choice of the RCSRR of major axis length *L_i_* and minor axis length *W_i_* in Figure 1b, the design equations for a desired notched band frequency *f_notch_* are given by:

(1) $$L_{i}+2W_{i}=\frac{\lambda_{g}}{2}=\frac{c}{2f_{notch}\sqrt{\varepsilon_{eff}}}$$

(2) $$\varepsilon_{eff}=\frac{\varepsilon_{r}+1}{2}+\frac{\varepsilon_{r}-1}{2}{\left(1+\frac{12h}{w_{f}}\right)}^{-0.5}$$

where *ε_r_* is the relative permittivity of the substrate, *λ_g_* is the guided wavelength at the desired frequency, *c* is the speed of light, and *f_notch_* is the desired notched frequency. The effective dielectric constant can be calculated from Equation (2), where *h* is the height of the substrate and *W_f_* is the width of the microstrip feedline. Parametric simulations have been performed to obtain the optimal width of the RCSRR. *W_s_* equals 0.3 mm.

For the initial choice of the RSRR of horizontal axis length *L_x_*, vertical axis length *L_y_* and width of the RSRR *W_s_* in Figure 1b, we can use Equation (3) to obtain the desired notch frequency for the resonators:

(3) $$2\left(L_{x}+L_{y}-2W_{s}\right)=\frac{\lambda_{g}}{2}=\frac{c}{2f_{notch(2)}\sqrt{\varepsilon_{eff}}}$$

where *f_notch_*_(2)_ is the desired resonant notched band frequency for the RSRR, and *λ_g_* is the guided wavelength at the desired frequency.

### 2.2. Structural Analysis

**Figure 3:**
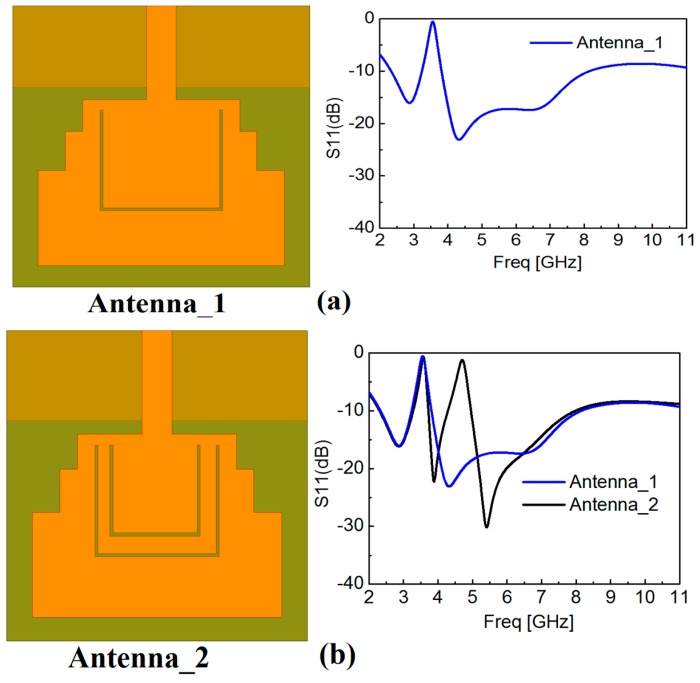

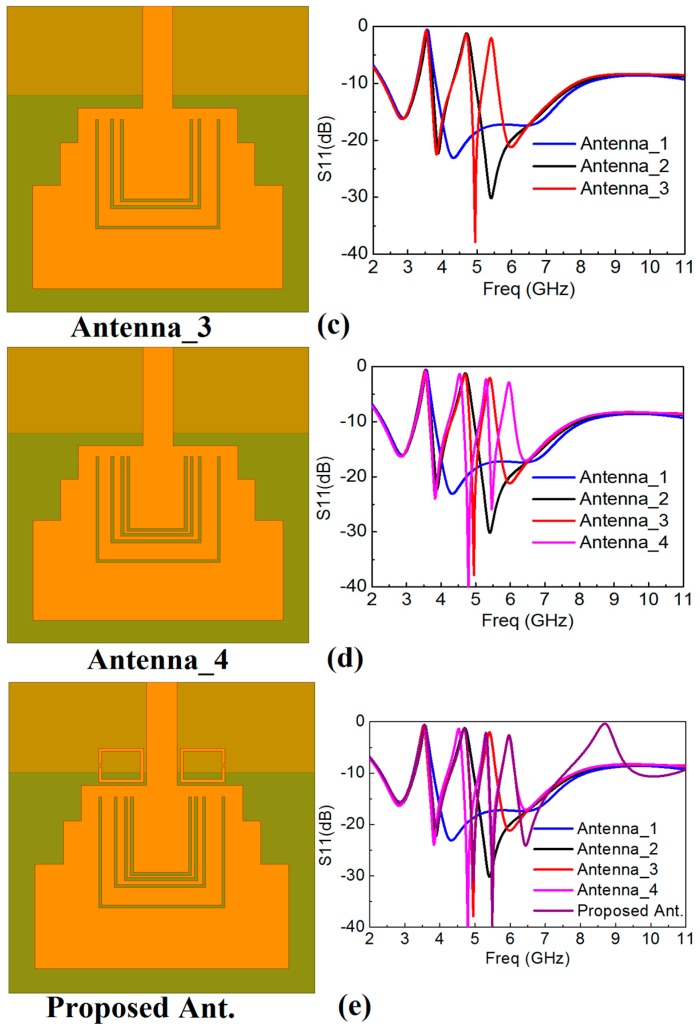
Design steps of the proposed antenna with the corresponding reflection coefficient results at each stage. The left column shows the antenna structure under design at different stages (**a**) Step 1: adding notch at Worldwide-Interoperability for Microwave-Access (WiMAX; 3.5 GHz); (**b**) Step 2: adding notch at Indian National Satellite (INSAT; 4.5 GHz); (**c**) Step 3: adding notch at the lower wireless local area network (WLAN; 5.25 GHz); (**d**) Step 4: adding notch at upper WLAN (5.7 GHz); (**e**) Step 5: adding notch at ITU 8 GHz (8.2 GHz).

Etching the slot resonators is one of the most effective techniques in designing the UWB antenna with multi-rejection bands without any size increase. After adequately calculating the initial value of the design variables for the band-notched frequency bands, it can be further optimized through parametric analysis and a step-by-step design procedure. To more clearly demonstrate the design process of this quintuple band-notched UWB antenna, Figure 3 presents the subsequent design process of the proposed quintuple band-notched UWB antenna and the corresponding frequency response at each sequence.

In the beginning, a single band-notched UWB antenna is designed to reject the WiMAX (3.5 GHz) frequency band. The structure of this antenna is shown in Figure 3a and termed as Antenna_1 which comprises of a single slot resonator using Equation (1), and optimized using the parametric analysis with HFSS. Then, a dual band-notched UWB antenna is designed by integrating another rectangular slot resonator within the first resonator. The dimensions of this resonator are also calculated from Equation (1) and further optimized at the center frequency of 4.5 GHz for notching the INSAT frequency band. This antenna along with its frequency response is shown in Figure 3b and termed as Antenna_2. Similarly, repeating the same procedure, we obtain the triple band-notched UWB antenna, and the quad band-notched UWB antenna termed as Antenna_3 and Antenna_4 by rejecting lower WLAN (5.25 GHz), and upper WLAN (5.7 GHz), respectively. Finally, a pair of RSRRs is placed in the vicinity of the feedline to obtain a broad notched frequency band at ITU 8.2 GHz (whose dimensions are calculated using Equation (3)), and optimized with HFSS. The final antenna is termed as the proposed antenna, and its frequency response is also presented in Figure 3e which shows the combined graph of all antennas and the variations within one plot.

This independent behavior of the slot resonator also makes the proposed antennas structure advantageous to be used for single, dual, triple quadruple and quintuple band rejection capabilities depending on the application requirements. By using the R-CSRR and RSRR, we can quickly set the multiple notches to the desired frequencies by selecting the length and width of the resonators according to Equations (1) and (3).

### 2.3. Simulated Input Voltage Standing Wave Ratio (VSWR) (Return–Loss) and Antenna Gain (Radiation Efficiency)

**Figure 4:**
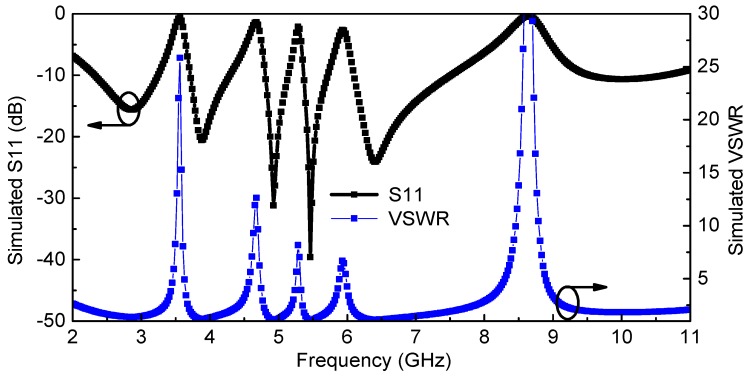
Simulated reflection coefficient and VSWR (Voltage Standing Wave Ratio) of the proposed quintuple band-notched UWB antenna.

**Figure 5:**
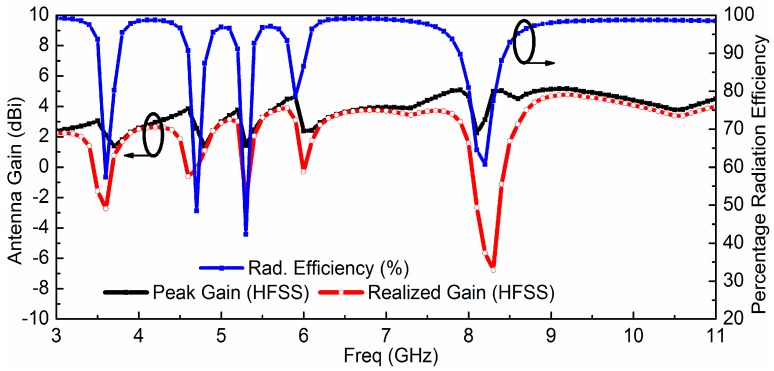
Simulated antenna gain from the radiator, percentage radiation efficiency, and realized antenna gain including the effects of the input mismatch of the quintuple band-notched UWB antenna.

The proposed quintuple band-notched UWB antenna is simulated using HFSS. Figure 4 shows the simulated reflection coefficient *Г_in_* and VSWR (Voltage Standing Wave Ratio) of the proposed antenna which reveals the effective filtering behavior at 3.5, 4.5, 5.25, 5.7, and 8.2 GHz for WiMAX, INSAT, lower WLAN, upper WLAN, and ITU 8 GHz frequency bands, respectively. The simulated antenna peak gain (*G*_0_), radiation efficiency (ƞ), and realized antenna gain (*G_r_ = G*_0_(1 − |*Г_in_*|^2^) are presented in Figure 5, which shows proper suppression occurs in the primary radiator represented by the antenna gain (*G*_0_) as well as at the antenna input in the form of the impedance mismatch (*Г_in_*) at the notched bands.

**Figure 6:**
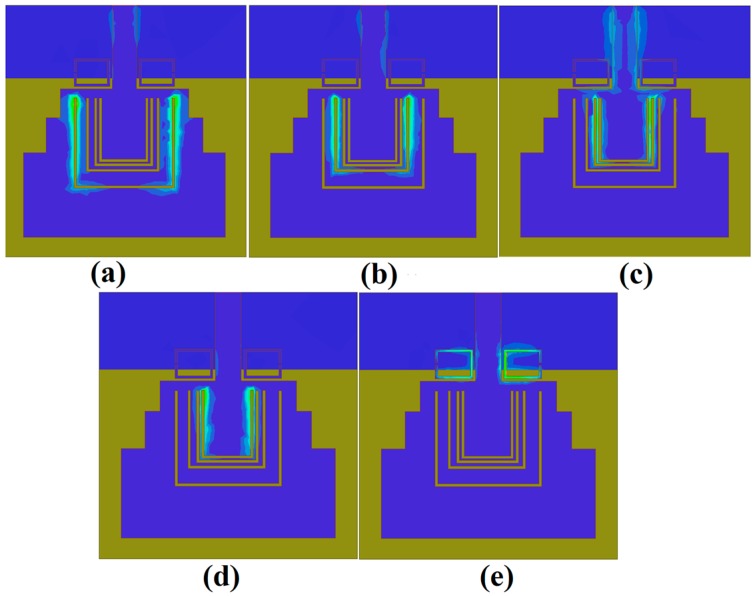
Surface current distributions of the proposed antenna at the corresponding center frequencies of the notched bands (**a**) 3.5 GHz; (**b**) 4.5 GHz; (**c**) 5.25 GHz; (**d**) 5.7 GHz; (**e**) 8.2 GHz.

To further evaluate the quintuple band-notching function resulting from R-CSRR and RSRR, the surface current distributions for the proposed antenna have been simulated at 3.5, 4.5, 5.25, 5.7, and 8.2 GHz and shown in Figure 6. It can be observed that the currents are mainly concentrated at these frequencies around the slot resonators which results in severe impedance mismatches around the resonant frequencies leading to the quintuple band-notching behavior of the antenna.

### 2.4. Parametric Analysis for the Optimal Width of the Resonators (W_s_)

**Figure 7:**
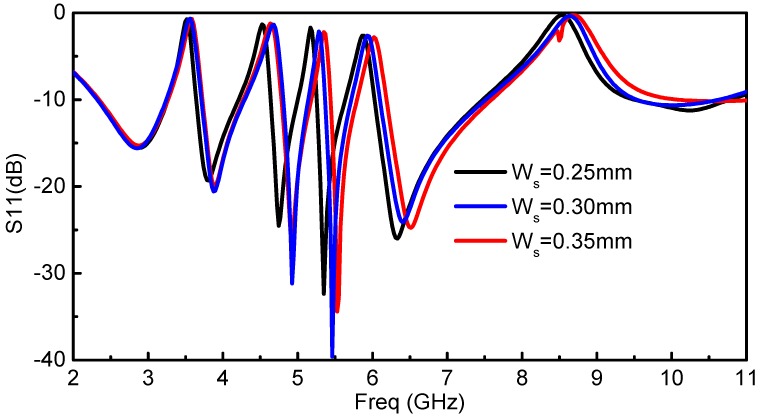
Effect of critical parameter *W_s_* on the response of the proposed antenna.

Due to the compact size of the proposed antenna with quintuple rejection bands, the reflection coefficient performance of the antenna is sensitive to geometrical parameters and requires appropriate optimizations and tuning. The parametric analysis has been carried out to illustrate the critical geometrical parameters of the proposed antenna that affect the notched frequency bands. The effect of *W_s_* (width of the R-CSRR and RSRR) is observed by varying its value, and the corresponding response is shown in Figure 7 by keeping all other design parameters to the fixed values. It is shown that by slightly varying *W_s_*, the corresponding notch frequency is shifted to the upper and lower frequency band, which is quite critical. After performing parametric tuning, the optimal resonator width has been selected as *W_s_* = 0.3 mm.

## 3. Measurement Results and Discussions

**Figure 8:**
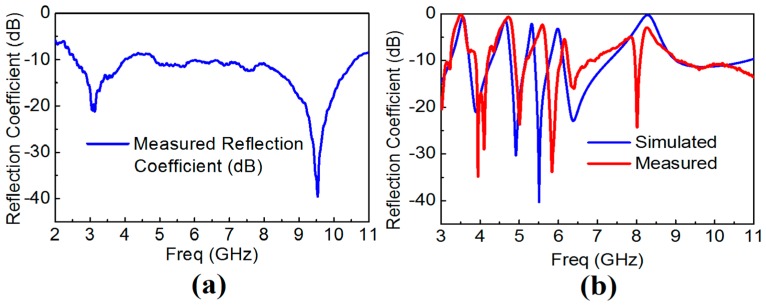
The measured frequency response of the reference and proposed quintuple band-notched UWB antenna (**a**) Measured reflection coefficient of the reference antenna; (**b**) Measured vs. simulated reflection coefficient of the proposed quintuple band-notched antenna.

**Figure 9:**
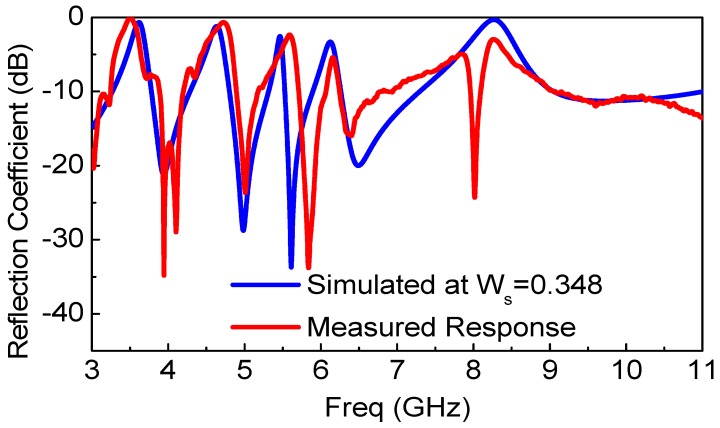
Simulated vs. measured frequency response of the proposed quintuple band-notched UWB antenna when *W_s_* = 0.348 mm in the simulation setup.

The reference and the proposed quintuple band-notched UWB antennas were fabricated, and their frequency response was measured to demonstrate the multiple notching responses. The Thru-Reflect-Line (TRL) calibration has been carried out after implementing the TRL calibration kit with the same antenna substrate and SMA connectors. The frequency response of both the reference and the proposed antennas after TRL calibration is shown in Figure 8. The response clearly reveals that there exists a filtering behavior centered at frequencies of 3.5, 4.5, 5.25, 5.7, and 8.2 GHz. While rejecting the WiMAX, INSAT, and ITU 8 GHz frequency bands as designed, there are slight frequency shifts towards the higher frequencies in the measurement. Table 2 presents the discrepancies between the simulated and measured notch frequencies. The minor frequency shifts were caused by the over-etched slot width from the milling machine. The slot width was measured using Vernier caliper at several different positions at the slot resonators. While we use *W_s_* equal to 0.3 mm, the measured mean and standard deviation values of *W_s_* were 0.3487 mm and 0.0258 mm, respectively. The frequency shifts in the lower (L-WLAN) and the upper WLAN (U-WLAN) frequency bands were relatively drastic where the mutual coupling between two slots was found to be larger than other slot resonators. The effect of the over-etched slot width (*W_s_*) becomes more dominant for L-WLAN and U-WLAN resonators as both the slot resonators are close to each other, which in turn has more significant mutual coupling, which shifts the resonance frequency much higher than other resonators. Figure 9 compares the simulated and measured reflection coefficient of the proposed antenna after setting *W_s_* = 0.3487 mm, which verifies our observation. Table 3 presents the discrepancy between the simulated and measurement response when the simulation setup is changed with *W_s_* = 0.348 mm, keeping all other parameters fixed.

**Figure 10:**
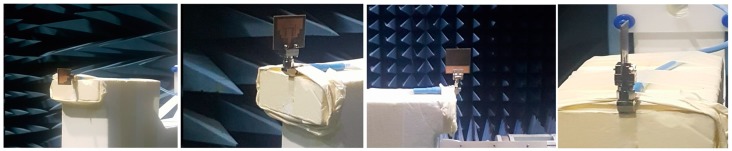
Radiation pattern measurement setup for the reference and proposed antenna at different orientations in the anechoic chamber.

**Figure 11:**
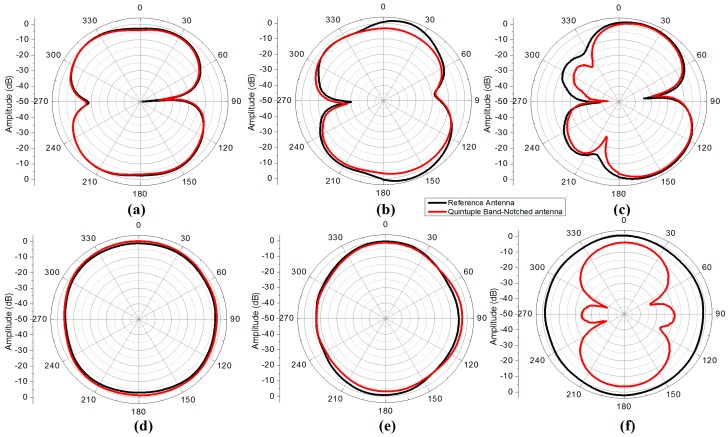
Measured radiation patterns of the reference vs. quintuple band-notched antenna (**a**) XZ plane (3.9 GHz); (**b**) XZ plane (6.1 GHz); (**c**) XZ plane (7.8 GHz); (**d**) YZ plane (3.9 GHz); (**e**) YZ plane (6.1 GH z); (**f**) YZ plane (7.8 GHz).

The radiation pattern of the reference and the proposed quintuple band-notched UWB antenna were measured in an anechoic chamber at three different frequencies which cover the center, lower, and upper frequencies of the entire UWB band. Figure 10 presents the measurement setup for the radiation pattern measurement of the reference and proposed antenna in an anechoic chamber. The chamber is equipped with the near-field planner scanner and a far-field tower to test and measure the radiation pattern of the antenna under test (AUT). At 3.9, 6.1, and 7.8 GHz the radiation patterns have been measured which cover the lower, upper, and mid frequencies of the UWB. The radiation pattern is measured at each 1 degree step interval in an anechoic chamber having a range of 0–360 °. The selected frequencies were 3.9, 6.1, and 7.8 GHz and its co-polarized beam pattern at the XZ-plane (E-plane) and YZ-plane (H-plane) are presented in Figure 11. There is a good agreement between the radiation pattern of the reference antenna and the proposed quintuple band-notched UWB antenna at 3.9 and 6.1 GHz, while the radiation pattern became distorted at 7.8 GHz and there arise nulls at both planes. It is due to the distortion in the phase distribution of the electric field and the increase in the magnitude of higher order harmonic modes at the higher frequency from the structure.

## 4. Comparison with Recently Proposed Designs

To validate the effectiveness of the proposed antenna over another state-of-the-art design, we perform a detailed comparison of the proposed antenna with other recently reported designs from the literature. Table 4 provides the comparison with recently proposed UWB antenna with multiple notches in terms of the dimensions of the antenna, and the number of notched bands. It is concluded that the presented antenna has obvious advantages in the antenna size with the largest number of rejection bands, a planar structure, and complete rejection of the interfering bands as well. Moreover, the proposed antenna in this paper also achieves stable radiation patterns over the complete UWB frequency band.

The proposed antenna is easy to fabricate and the notched frequencies can be adjusted with the width of the slot resonators as compared to other antennas mentioned in the table which utilize irregular patch geometry, irregular slot resonators on the patch and ground plane, multilayered structures for notching, and parasitic stubs which lead to complex structures which result in a difficulty in massive fabrication. It is noteworthy that the implemented antenna in [19,20,21,22,23,24,25,26,27,28,29] completely rejected overall 5/6 GHz WLAN bands, which led to uncompromising signal quality degradation of the received information. The antenna will be highly applicable in microwave imaging sensing applications, motion sensing, and high speed wireless communication systems including wireless sensor networks, mobile handsets, and IoT due to its small size with an excellent performance as compared with other state-of-the-art structures.

## 5. Application of the Proposed Antenna to the Breast Tumor Sensing

**Figure 12:**
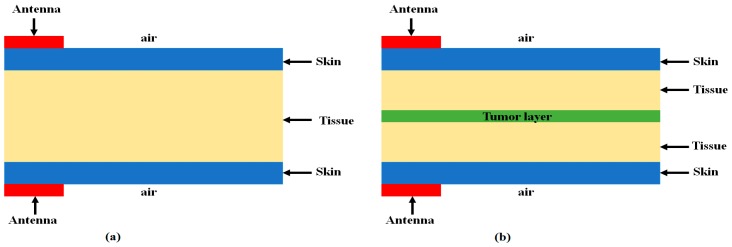
Breast phantom model developed for investigation of tumor cell (cross view). (**a**) Breast phantom without tumor; (**b**) Breast phantom with tumor.

Owing to the integrated quintuple notched bands for wireless sensor networks, mobile handsets, and the Internet of things (IoT), the proposed compact antenna can be applicable to various portable UWB sensors without severe interruption with aforementioned wireless system. The feasibility of the proposed antenna is investigated in detecting tumor cells in a canonical breast phantom model using the 3D EM simulator, HFSS. As presented in Figure 12, the proposed band-notched UWB antenna is placed at each side of the biological breast phantom model within the frequency range of 3–11 GHz. The model is an inhomogeneous dielectric medium to simulate the human breast by including skin, tissue, and tumor. The breast model utilized in our simulation setup with and without tumor are shown in Figure 12a,b, respectively. The permittivity and conductivity of the skin, tissue, and tumor are given in Table 5, calculated from [42,43,44]. The breast tissue layer has been covered with two skin layers, one at the top side and another at the bottom side. The stacked organic layers were scanned by two proposed quintuple notched antennas (one transmitting and the other receiving) with and without tumor cell.

**Figure 13:**
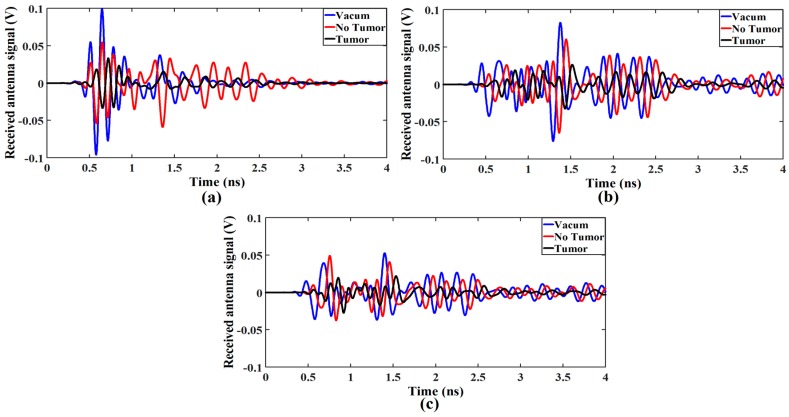
Received waveform responses for (**a**) Both antennas are placed in contact with stacked organic layers, and each antenna is placed (**b**) 5 mm away and (**c**) 10 mm from the stacked organic layers.

After transmitting the UWB pulses with the proposed antenna, the received antenna waveform response is used to detect the tumor cells by comparing the transmitted waveform with and without tumor cells. First, both antennas are placed in a vacuum without placing any stacked layer, and the received waveform is observed. Then, stacked organic layers with and without tumor layer are placed between transmitted and receiving antennas, and the waveform is observed. The combined received waveforms observed in above three cases are presented in Figure 13.

It is seen that the amplitude of the received waveform decreases considerably in case of the tumor layer as shown in Figure 13a, which may lead the conclusion that defected cells are present in the body. The same process is repeated by shifting each antenna 5 and 10 mm away from the stacked organic layers, and the received waveform is observed as presented in Figure 13b,c, respectively. It is clear from Figure 13b,c that moving the antennas away from the stacked biological organic layer degrades overall signal strength. However, the amplitude of the received waveform for the stacked layers with the tumor drops more considerably. It is due to the fact that the existence of the stacked organic layers such as skin and tissue produces multiple reflections and losses on the surface of each layer during the pulse transmission. Notably, the presence of defected tumor layer within the body increases the energy losses apparently because of the different dielectric properties of the tumor cells as compared to skin and tissue layers.

## 6. Conclusions

In this paper, we presented a microstrip-fed ultra-wideband (UWB) antenna with quintuple rejection bands for wireless sensor networks. Quintuple rejection bands were realized at the frequencies of 3.5, 4.5, 5.25, 5.7, and 8.2 GHz utilizing four R-CSRRs on the radiating patch and placing two RSRRs near the feedline-patch junction of the conventional UWB antenna. The design guidelines of the implemented notched bands are discussed with corresponding equations. The width of the slot resonator has been found to be an essential tuning knob to achieve the desired notching frequencies. With its compact size, the measured results of the proposed antenna correspond well with the simulation. The antenna possesses a nearly omnidirectional radiation pattern with an excellent gain suppression at the notched-bands over the entire frequency band. The proposed antenna is also evaluated to detect tumor cells in the breast phantom model. Hence, the proposed antenna will be the most suitable candidate to be used in microwave imaging sensing applications, wireless sensor networks, and IoT applications.

## Figures and Tables

**Table 1:** Dimensions of the reference and proposed antennas.

Parameter	Value (mm)	Parameter	Value (mm)	Parameter	Value (mm)	Parameter	Value (mm)
L_sub_	30	L_2_	4.3	W_1_	25	W_7_	6.7
L_gnd_	9.0	L_3_	3.5	W_2_	2.75	W_8_	5.9
W_sub_	29	L_4_	11	W_3_	1.75	W_s_	0.30
W_f_	3.0	L_5_	9.0	W_4_	6.5	L_x_	3.4
L_f_	10.3	L_6_	8.4	W_5_	12.4	L_y_	4.4
L_1_	10.5	L_7_	7.8	W_6_	9.0	g_1_	0.3

**Table 2:** Discrepancies between the simulated and measured results.

	WiMAX	INSAT	LWLAN	UWLAN	ITU 8 GHz
Simulated (f_1_)	3.54	4.65	5.31	5.90	8.26
Measured (f_2_)	3.57	4.70	5.51	6.15	8.28
Shift in frequency (∆f = f_2_ − f_1_)	0.03	0.05	0.20	0.25	0.02

**Table 3:** Discrepancies between the simulated and measured results when simulated with *W_s_* = 0.348 mm.

	WiMAX	INSAT	LWLAN	UWLAN	ITU 8 GHz
Simulated (f_1_)	3.60	4.66	5.43	6.14	8.27
Measured (f_2_)	3.57	4.70	5.51	6.15	8.28
Shift in frequency (∆f = f_2_ − f_1_)	0.03	0.04	0.08	0.01	0.01

**Table 4:** Performance comparison of the proposed antenna with another recently reported state-of-the-art design.

Literature	Size (mm)	Filtering Bands (GHz)	Remarks
[18]	40 × 30	3.3–3.8	Antenna reject WiMAX and complete WLAN frequency bands
		5.0–6.0	
[19]	26 × 24	5.1–5.9	Antenna reject the complete WLAN band
[22]	45 × 50	5.1–5.95	Antenna rejects complete WLAN band
[23]	36 × 22	5.1–5.9	Antenna reject the complete WLAN band with Large size and irregular structure
[25]	25 × 20	3.3–3.8	Antenna rejects the WiMAX and complete WLAN band with stair cased ground plane
		5.1–5.85	
[26]	33 × 28	3.4–3.9	Rejects WiMAX and upper WLAN bands with three different CSRR pairs
		5.6–5.95	
[27]	28 × 15	3.0–3.9	Extra band rejection and irregular geometry at the cost of size reduction
		4.9–5.8	
[32]	36 × 34	3.31–3.90	Triple band rejection with different irregular resonators placements
		5.20–5.35	
		5.8–6.0	
[33]	34.6 × 24	3.1–3.9	Extra rejection bands using three different pairs of circular and rectangular CSRRs
		4.5–5.35	
		5.75–6.0	
[34]	40 × 40	3.1–3.8	Triple band rejection at the cost of increasing size
		5.0–6.0	
		7.9–8.7	
[35]	40 × 40	2.37–2.39	Quad notching at the cost of increased size Extra band-notching for WiMAX and X-band satellite communication while complete WLAN band-notching
		3.27–3.76	
		5.2–5.89	
		8.06–8.80	
[36]	33.5 × 30	3.30–3.70	Quad notching using multilayered structures Notching is not deep enough for good rejection Antenna structure is complex
		4.50–4.80	
		5.10–5.35	
		5.75–5.95	
[38]	30 × 28	3.30–3.36	Quad notching using CSRR structures
		4.50–4.70	
		5.15–5.35	
		5.70–5.825	
[39]	80 × 80	1.85–2.49	Quintuple band-notching with extra rejection bands Size of the antenna is relatively large
		2.91–3.40	
		3.95–5.39	
		5.65–6.25	
		7.19–8.78	
[41]	31 × 25	3.40–3.80	Compact Quintuple band-notched UWB antenna utilizing different slot resonators Irregular placement of the resonators on the patch and ground plane
		5.1–5.35	
		5.60–6.0	
		7.15–7.65	
		8.05–8.65	
This Work	28 × 30	3.25–3.60	Compact Quintuple band notching with a simple structure and regularly shaped resonators
		4.40–4.80	
		5.10–5.40	
		5.75–5.95	
		7.50–8.75	

**Table 5:** Dielectric properties of the skin, tissue, and tumor.

Type	Dielectric Constant	Conductivity (S/m)	Mass Density (kg/m^3^)
Skin	36	4	1010
Tissue	50	7	1040
Tumor	67	5	1040
